# Crude glycerin is an efficient alternative to corn in the diet of feedlot lambs

**DOI:** 10.5194/aab-64-387-2021

**Published:** 2021-09-17

**Authors:** Fernada Almeida Merlim, Américo Garcia Silva Sobrinho, Thiago Henrique Borghi, Luís Gabriel Alves Cirne, Roberta Lima Valença, Fabiana Alves Almeida, Viviane Endo, Carlos Renato Viegas, Nivea Maria Brancacci Lopes Zeola

**Affiliations:** 1 Department of Animal Science, São Paulo State University, Jaboticabal, São Paulo, 14884-900, Brazil; 2 Institute of Biodiversity and Forestry, Federal University of Western Pará, Santarém, Pará, 68035-110, Brazil

## Abstract

The objective of this study was to evaluate the intake, digestibility, nitrogen balance, and performance of feedlot lambs fed diets containing crude glycerin. A total of 30 Ile de France lambs were confined to individual pens at an average age of 45 d: 15.1 kg initial body weight and 32.2 kg final body weight. The animals were distributed in a completely randomized design and fed three diets containing fresh sugarcane as forage (50 %) and concentrate (50 %), with or without the inclusion of 100 and 200 g vegetable crude glycerin per kilogram dry matter (DM) replacing corn. Dietary glycerin inclusion reduced the intake of ether extract (P<0.001) and total carbohydrates (P=0.048) as well as the apparent digestibility of ether extract (P<0.001), but it had no effect on the intake and digestibility of the other nutrients nor on lamb performance. The apparent nitrogen balance of lambs on the three diets was positive. Although it does not affect the intake and digestibility of most nutrients, the inclusion of 100 and 200 $g\;{\mathrm{kg}}^{-1}\;\mathrm{DM}$ of crude glycerin in the diet tends to worsen lamb performance, indicating that the ideal level of inclusion should be below 100 $g\;{\mathrm{kg}}^{-1}\;\mathrm{DM}$ of crude glycerin.

## 1 Introduction

In view of the increasing worldwide demand for animal protein, including sheep meat, animals need to be produced on a large enough scale to meet the requirements of the consumer market. Nutrition plays a fundamental role in animal production. In this respect, nutritional strategies should be adopted to incorporate waste and by-products of agricultural industries into the feed base of ruminants in order to reduce the use of grains in animal feed, thereby leaving them for human consumption. Moreover, in the current economic scenario, grains have reached high prices, rendering diets more expensive and increasing the costs of animal production. For this reason, by-products of the biodiesel industry, such as vegetable crude glycerin, have been studied for use in place of traditional feed ingredients (Gunn et al., 2010; Lage et al., 2010).

The inclusion of crude glycerin in diet formulations provides a gluconeogenic substrate for ruminants, as these animals use the glycerol in dietary glycerin to form glucose (Krehbiel, 2008). In this way, this feedstuff can partially replace starchy feedstuffs in diets, especially cereal grains such as corn. However, optimal replacement formulations are yet to be established.

Gunn et al. (2010) evaluated the effects of up to 450 $g\;{\mathrm{kg}}^{-1}$ crude glycerin in dry matter (DM) on lamb fatness and found that the inclusion of 150 $g\;{\mathrm{kg}}^{-1}$ glycerin in the diet did not compromise animal performance. Lage et al. (2010) observed decreased performance in lambs fed up to 120 $g\;{\mathrm{kg}}^{-1}$ crude glycerin in DM. Oliveira Filho et al. (2016) studied the inclusion of up to 108 $g\;{\mathrm{kg}}^{-1}$ crude glycerin in DM of sugarcane silage and found no changes in intake, digestibility, or nitrogen balance in lambs. Ribeiro et al. (2018) evaluated the use of up to 210 $g\;{\mathrm{kg}}^{-1}$ crude glycerin in DM in lamb diets and reported that the inclusion of 47 $g\;{\mathrm{kg}}^{-1}$ of the ingredient provided the greatest increase in body growth.

Sugarcane is one of the most commonly used forages by farmers due to characteristics such as high yield potential and lower production costs. Thus, the present study was motivated by the fact that no studies have examined the performance of lambs fed diets containing crude glycerin as a substitute for corn and using fresh sugarcane as the roughage component. In this context, this study investigates the effects of replacing dietary corn with two concentrations of glycerin on the intake, digestibility, nitrogen balance, and performance of feedlot lambs.

## 2 Material and methods

### 2.1 Location and ethical considerations

The experiment was conducted in the city of Jaboticabal, state of São Paulo, Brazil (21${}^{\circ }$14${}^{\prime }$05${}^{\prime \prime }$ S, 48${}^{\circ }$17${}^{\prime }$09${}^{\prime \prime }$ W; 615.01 ma.s.l.; atmospheric pressure of 944.3 hPa; average temperature of 22.2 ${}^{\circ }C$; relative humidity of 72.5 %; precipitation of 1453.4 mm), following the guidelines set by the Ethics Committee on Animal Use (approval no. 005962/12).

### 2.2 Animals, experimental design, and diets

A total of 30 newly weaned 45 d old uncastrated male Ile de France lambs with a body weight of 15±0.2 kg were individually identified, dewormed, and housed in individual 1.0 $m^{2}$ pens in a shed with a suspended slatted floor, equipped with feeders and drinkers. A total of 10 lambs were randomly allocated to each of the three dietary treatments.

**Table 1:** Ingredients and chemical composition of experimental diets.

Composition	Crude glycerin		
	($g\;{\mathrm{kg}}^{-1}\;\mathrm{DM}$)		
	0	100	200
Ingredient proportions ($g\;{\mathrm{kg}}^{-1}\;\mathrm{DM}$)			
Sugarcane	500.0	500.0	500.0
Crude glycerin	0.0	100.0	200.0
Corn grain, ground, dry	254.3	136.9	10.4
Soybean meal	217.7	240.0	265.5
Dicalcium phosphate	2.8	2.2	1.8
Calcium carbonate	5.7	4.3	3.3
Mineral–vitamin supplement${}^{a}$	9.5	9.5	9.5
Urea	10.0	10.0	10.0
Chemical composition ($g\;{\mathrm{kg}}^{-1}\;\mathrm{DM}$)			
Dry matter ($g\;{\mathrm{kg}}^{-1}$ as fed)	381.7	359.6	345.8
Mineral matter	53.4	67.2	81.7
Crude protein	165.3	167.4	169.0
Ether extract	19.6	11.0	6.3
Neutral detergent fiber	293.7	295.3	290.4
Acid detergent fiber	123.4	131.7	120.5
Total carbohydrates	788.7	759.8	721.1
Nonfibrous carbohydrates	563.5	528.1	517.4
Gross energy ($\mathrm{Mcal}\;{\mathrm{kg}}^{-1}\;\mathrm{DM}$)	3.88	3.99	3.72

${}^{a}$ Guaranteed levels per kilogram of product: 120 g Ca, 90 g Cl, 62 g Na, 54 g Mg, 50 g P, 34 g S, 1600 mg Zn, 1500 mg Mn, 1064 mg Fe, 730 mg F (Max), 50 mg Cu, 25 mg I, 20 mg Se, 10 mg Co, 100 000 IU vitamin A, 40 000 IU vitamin D3, and 600 IU vitamin E.

**Table 2:** Percentage composition of fatty acids in diets.

Fatty acid (%)	Crude glycerin		
	($g\;{\mathrm{kg}}^{-1}\;\mathrm{DM}$)		
	0	100	200
Saturated			
Capric (C10:0)	0.04	0.03	0.03
Lauric (C12:0)	0.11	0.10	0.17
Myristic (C14:0)	0.26	0.23	0.39
Pentadecanoic (C15:0)	0.11	0.07	0.10
Palmitic (C16:0)	17.21	19.08	22.98
Margaric (C17:0)	0.20	0.15	0.20
Stearic (C18:0)	4.36	4.68	4.27
Arachidic (C20:0)	0.87	0.77	0.73
Behenic (C22:0)	0.35	0.38	0.49
Tricosanoic (C23:0)	0.14	0.14	0.15
Lignoceric (C24:0)	0.60	0.54	0.54
Monounsaturated			
Palmitoleic (C16:1)	1.40	1.87	0.91
Heptadecenoic (C17:1)	0.07	0.08	0.11
Oleic (C18:1ω9)	31.61	31.19	30.10
*Cis*-vaccenic (C18:1ω7)	0.98	0.88	0.93
Eicosenoic (C20:1ω9)	0.39	0.37	0.38
Polyunsaturated			
Linoleic (C18:2ω6)	38.82	36.65	35.07
α-Linolenic (C18:3ω3)	2.19	2.56	2.18
γ-Linolenic (C18:3ω6)	0.04	0.04	0.03
Eicosadienoic (C20:2)	0.21	0.16	0.21
Eicosatrienoic (C20:3ω6)	0.04	0.03	0.03
Omega-3${}^{a}$ (ω3)	2.19	2.56	2.18
Omega-6${}^{b}$ (ω6)	38.90	39.72	35.13
Omega-6:Omega-3 (ω6:ω3)	17.76	15.51	16.11

DM denotes dry matter. ${}^{a}$ ω3=(C18:3ω3); ${}^{b}$ ω6=(C18:2ω6+C18:3ω6+C20:3ω6) (Borghi et al., 2016).

The tested diets were formulated with or without the inclusion of vegetable crude glycerin at 100 or 200 $g\;{\mathrm{kg}}^{-1}\;\mathrm{DM}$ replacing corn (Tables 1, 2). Sugarcane variety IAC 86-2480 (developed by the Agronomic Institute of Campinas, Brazil, for use in animal feed due to the low amount of fiber compared with other varieties) was chopped into 1.0 cm particles and supplied fresh. The glycerin from soybean oil (83.90 % glycerol, 12.31 % moisture, and 3.79 % salts) was weighed and mixed with the concentrate at the time the diets were supplied to the lambs. The resulting diets had similar protein (16 %) and energy (3.35 Mcal ${\mathrm{kg}}^{-1}\;\mathrm{DM}$ metabolizable energy) contents and were prepared as recommended by the National Research Council (NRC, 2007) requirements for weaned lambs, for an estimated 250 $g\;d^{-1}$ weight gain. The roughage : concentrate ratio was 50:50, and the diets were provided ad libitum (at least 10 % leftovers) at 07:00 and 17:00 h.

### 2.3 Chemical analysis

The chemical composition of the diets (Table 1) is presented in grams per kilogram of dry matter ($g\;{\mathrm{kg}}^{-1}\;\mathrm{DM}$) and was determined after oven-drying at 65 ${}^{\circ }C$ for 24 h and milling through a 1.0 mm screen (AOAC, 1998; ID 934.01). Mineral matter (MM) was measured after combustion at 550 ${}^{\circ }C$ for 16 h (AOAC, 1998; ID 942.05). The nitrogen (N) concentration was obtained using the Kjeldahl method (AOAC, 1998; ID 988.05); crude protein (CP) was calculated as the N content multiplied by the factor 6.25; and the ether extract (EE) content was obtained using method ID 920.39 (AOAC, 1998). Neutral detergent fiber (NDF) and acid detergent fiber (ADF) contents were measured according to the recommendations of Van Soest (1994): using bags (F57) and the ANKOM 200 device (ANKOM Technology Corp., Fairport, NY, USA) with heat-stable α-amylase. Total carbohydrates (TC) were estimated by following the procedure of Sniffen et al. (1992). Nonfibrous carbohydrates (NFC) were calculated as the difference between TC and NDF (Sniffen et al., 1992). Gross energy (GE) was obtained by sample combustion in an adiabatic bomb calorimeter (PARR Instruments). Lipid extraction was performed as described by Bligh and Dyer (1959) to determine the fatty acid profile. The extracted lipids were converted to fatty acid methyl esters following the technique of Hartman and Lago (1973) and were analyzed using a Shimadzu 14B gas chromatograph equipped with a flame ionization detector and fused silica capillary column (OMEGAWAX250, size: 30m×0.25mm×0.25 µm, cat. no. 24136 Supelco). The injector and detector temperatures were 250 and 280 ${}^{\circ }C$, respectively, and the helium flow ratio was 1 $\mathrm{mL}\;\min^{-1}$. After injection (1 µL), the column temperature was held at 100 ${}^{\circ }C$ for 2 min and then increased to 220 ${}^{\circ }C$ at 4 ${}^{\circ }C\;\min^{-1}$. Following this, the temperature was kept at 220 ${}^{\circ }C$ for 25 min. The peaks were identified by comparing retention times with the standards for fatty acid methyl esters from Sigma.

### 2.4 Intake, apparent nutrient digestibility, and nitrogen balance

Leftovers were collected and weighed daily before the morning feeding to determine dry matter intake and adjust the feed supply. The intake of nutritional components was estimated by calculating the difference between the total of each nutrient contained in the feed offered to the lambs and the total of each nutrient in the leftovers.

A total of 45 d after the beginning of the performance trial, the digestibility trial was started using 15 lambs (5 in each treatment; the choice of lambs was random) from the same 30 lambs, with an average body weight of 23 kg (Endo et al., 2015). The lambs were placed in metabolic cages that allowed for the separate collection of feces and urine, where they remained for 7 d of adaptation followed by 5 d of total feces and urine collection. The experiment was laid out in a completely randomized design with three treatments and five replicates. After this period, the lambs returned to the performance trial in the feedlot.

Feces were collected in plastic basins, and urine was collected in plastic buckets containing 100 mL of 20 % sulfuric acid solution ($H_{2}{\mathrm{SO}}_{4}$) to acidify it and avoid ammonia loss by volatilization; this volume was later deducted from the total daily urine volume. Plastic buckets were cut in a bevel shape and covered with fine mesh so that the feces falling on the screen would slide into the bowl and be separated from the urine. Ten percent of the feces and urine was sampled and stored at -18 ${}^{\circ }C$ daily, and the samples were used to form one composite sample per lamb at the end of the digestibility trial.

Apparent nutrient digestibility (AND) was calculated using the following equation: AND = [(kilograms of the portion ingested - kilograms of the portion excreted)/(kilograms of the portion ingested)] × 100.

Apparent nitrogen balance (ANB) was calculated using the following formulae: $\text{ANB or }N_{\text{retained}}=N_{\text{intake}}-(N_{\text{feces}}+N_{\text{urine}});N_{\text{absorbed}}=N_{\text{intake}}-N_{\text{feces}}$; and $N_{\text{intake}}=N_{\text{supplied}}-N_{\text{orts}}$, and expressed in grams per day ($g\;d^{-1}$) and in grams per metabolic weight per day.

### 2.5 Performance

Feed conversion was calculated as the ratio between DM intake (kg) and average weight gain (kg). To determine weight gain, the animals were weighed at the beginning of the experiment and then every 7 d (in the morning, before feed supply) until the final body weight (BW) measurement (32.2±0.2 kg) when the lambs were slaughtered. Average daily gain (ADG) was calculated as the ratio between total BW gain and the duration of the experimental period.

### 2.6 Statistical analysis

The experiment was laid out in a completely randomized design with three treatments (0, 100, and 200 $g\;{\mathrm{kg}}^{-1}\;\mathrm{DM}$ of crude glycerin) and 10 replicates each. Results were assessed using analysis of variance and regression, with degrees of freedom decomposed into linear or quadratic effects, according to the percentages of crude glycerin. The significance of the regressions was obtained by an F test at the 1 % or 5 % probability levels using the MIXED procedure of Statistical Analysis System (SAS) version 9.1 (SAS, 2009).

## 3 Results

The inclusion of crude glycerin in the lambs' diets reduced (P<0.05) the intake of ether extract (EE) 19.3, 10.3, and 4.5 g and total carbohydrates (TC) 720.0, 656.3, and 576.8 g to 0, 100, and 200 $g\;{\mathrm{kg}}^{-1}\;\mathrm{DM}$ of crude glycerin, respectively. However, there was no effect on the intake of other nutrients (Table 3). Apparent nutrient digestibility was also not altered by the different diets, except for EE digestibility (P<0.001), which was reduced (841.2, 736.4, and 482.6 $g\;{\mathrm{kg}}^{-1}$) with the increasing levels of dietary crude glycerin (Table 3).

**Table 3:** Intake and digestibility of nutrients of feedlot lambs fed diets containing crude glycerin.

Variable	Crude glycerin ($g\;{\mathrm{kg}}^{-1}\;\mathrm{DM}$)			SEM${}^{a}$	p value${}^{b}$	
	0	100	200		L	Q
	n=10	n=10	n=10			
Intake (g)						
Dry matter	926.0	883.0	821.3	32.39	0.225	0.896
Mineral matter	51.3	61.5	69.8	4.14	0.080	0.905
Crude protein	160.5	154.0	142.5	6.90	0.335	0.874
Ether extract${}^{c}$	19.3	10.3	4.5	1.91	<0.001	0.243
Neutral detergent fiber	243.5	221.8	215.5	7.56	0.154	0.631
Acid detergent fiber	95.8	93.5	93.3	3.63	0.804	0.909
Total carbohydrates${}^{d}$	720.0	656.3	576.8	29.08	0.048	0.888
Nonfibrous carbohydrates	535.3	489.8	435.0	22.55	0.081	0.919
Apparent digestibility of nutrients ($g\;{\mathrm{kg}}^{-1}$)	n=5	n=5	n=5			
Dry matter	783.3	775.6	795.2	0.57	0.409	0.283
Crude protein	752.9	739.6	754.4	0.71	0.944	0.403
Ether extract${}^{e}$	841.2	736.4	482.6	4.91	<0.001	0.122
Neutral detergent fiber	558.0	531.2	571.8	1.71	0.762	0.402
Acid detergent fiber	402.7	409.8	457.8	3.47	0.563	0.802
Total carbohydrates	809.0	797.4	807.2	0.51	0.892	0.374
Nonfibrous carbohydrates	922.5	902.1	928.6	0.61	0.672	0.081

${}^{a}$ SEM is the standard error of the mean. ${}^{b}$ The effects are denoted as follows: L – linear and Q – quadratic. ${}^{c}$ Y=18.708-0.737x, $r^{2}=0.98$. ${}^{d}$ Y=722.625-7.162x, $r^{2}=0.99$. ${}^{e}$ Y=86.601-1.792x, $r^{2}=0.94$. n denotes the number of lambs per treatment.

The apparent nitrogen balance of the lambs on the three diets was positive and not significantly (P>0.05) different between groups (Table 4). Dietary crude glycerin also had no effect (P>0.05) on nitrogen intake, absorbed nitrogen, or nitrogen excreted in urine and feces.

**Table 4:** Apparent nitrogen balance (ANB) in feedlot lambs fed diets containing crude glycerin.

Variable	Crude glycerin ($g\;{\mathrm{kg}}^{-1}\;\mathrm{DM}$)			SEM${}^{a}$	p value${}^{b}$	
	0	100	200		L	Q
	n=5	n=5	n=5			
N intake						
Grams per animal per day	25.2	24.7	22.8	1.11	0.434	0.808
Grams per metabolic weight per day	2.3	2.3	2.1	0.10	0,438	0.766
Fecal N						
Grams per animal per day	6.4	6.5	5.5	0.35	0.346	0.513
Grams per metabolic weight per day	0.6	0.6	0.5	0.03	0.360	0.464
Grams per kilogram N intake	252.7	260.4	242.2	0.86	0.650	0.522
Urinary N						
Grams per animal per day	0.8	0.8	0.8	0.06	0.913	0.879
Grams per metabolic weight per day	0.1	0.1	0.1	0.01	0.861	0.919
Grams per kilogram N intake	33.3	33.4	34.9	0.27	0.829	0.916
Urinary N						
Grams per animal per day	19.3	18.2	17.3	0.85	0.390	0.940
Grams per metabolic weight per day	1.8	1.7	1.6	0.08	0.392	0.972
Grams per kilogram N intake	76.9	74.0	75.8	1.03	0.684	0.320
Retained N or ANB						
Grams per animal per day	18.0	17.4	16.6	0.83	0.522	0.967
Grams per metabolic weight per day	1.7	1.6	1.5	0.08	0.518	0.929
Retained N/N intake	71.4	70.6	72.3	0.84	0.696	0.537
Retained N/Absorbed N	93.8	95.4	95.4	0.56	0.277	0.503

${}^{a}$ SEM is the standard error of the mean. ${}^{b}$ The effects are denoted as follows: L – linear and Q – quadratic. n denotes the number of lambs per treatment.

**Table 5:** Performance of feedlot lambs fed diets containing crude glycerin.

Variable	Crude glycerin ($g\;{\mathrm{kg}}^{-1}\;\mathrm{DM}$)			SEM${}^{a}$	p value${}^{b}$	
	0	100	200		L	Q
	n=10	n=10	n=10			
Initial body weight (kg)	15.12	15.02	15.12	0.03	0.998	0.242
Final body weight (kg)	32.32	32.18	32.24	0.04	0.382	0.211
Days in feedlot	68	74	78	2.80	0.370	0.895
DM intake						
Kilograms per day	0.703	0.642	0.613	0.02	0.109	0.740
Percent BW	2.99	2.74	2.60	0.07	0.111	0.766
Grams per metabolic weight per day	65.79	60.23	57.33	1.45	0.112	0.761
ADG${}^{c}$ (kg)	0.264	0.233	0.221	0.01	0.193	0.744
FC${}^{d}$ (kg DM ${\mathrm{kg}}^{-1}$ weight gain)	2.78	2.80	2.82	0.09	0.904	0.981

${}^{a}$ SEM is the standard error of the mean. ${}^{b}$ The effects are denoted as follows: L – linear and Q – quadratic. ${}^{c}$ ADG is the average daily gain. ${}^{d}$ FC is the feed conversion. n denotes the number of lambs per treatment.

The lambs performed equally well on diets supplemented with crude glycerin when compared with the controls (P>0.05), with ADGs of 0.264, 0.233, and 0.221 kg consuming approximately 0.703, 0.642, and 0.613 $\mathrm{kg}\;d^{-1}\;\mathrm{DM}$ to 0, 100, and 200 $g\;{\mathrm{kg}}^{-1}\;\mathrm{DM}$ of crude glycerin, respectively (Table 5). This intake level represented 2.99 %, 2.74 %, and 2.60 % of total BW and a feed conversion of 2.78, 2.80, and 2.82 with the increasing levels of dietary crude glycerin. Although not statistically different (P>0.05), the feedlot days were 68 d for the lambs that did not receive crude glycerin in their diet, 74 d for the lambs that received 100 $g\;{\mathrm{kg}}^{-1}\;\mathrm{DM}$, and 78 d for the lambs that received 200 $g\;{\mathrm{kg}}^{-1}\;\mathrm{DM}$ (Table 5).

## 4 Discussion

The reduction in the intake and digestibility of EE and the reduction in the intake of TC in the animals fed crude glycerin are directly related to the decreasing concentration of corn in the diets, as glycerin does not contribute to EE and has a lower carbohydrate content (Table 1). In the study of Ribeiro et al. (2018), the authors included 70, 140, and 210 $g\;{\mathrm{kg}}^{-1}\;\mathrm{DM}$ of crude glycerin in the diet of lambs and observed a decrease in the intake of NDF and nonfibrous carbohydrates (NFC) and an increase in EE intake. Saleem and Singer (2017) reported a quadratic reduction in the DM intake and a quadratic increase in the DM and EE digestibility of lambs fed with 0 %, 5 %, and 10 % of glycerol replacing corn in their diet. Lage et al. (2010) investigated the inclusion of 0, 30, 60, 90, and 120 $g\;{\mathrm{kg}}^{-1}\;\mathrm{DM}$ of crude glycerin in the diet of feedlot lambs and also reported the influence of glycerin inclusion. In their experiment, there was a 30 % reduction in voluntary DM intake following the addition of 120 $g\;{\mathrm{kg}}^{-1}$ dietary crude glycerin, which resulted in lower intake of organic matter (OM), CP, NDF, and NFC.

Therefore, why are there differences in the recommendations for crude glycerin inclusion in lamb diets? Perhaps the answer lies in the type of glycerin used. Glycerin has variable levels of glycerol, water, methanol, and fatty acids and is classified as either low purity (50 % to 70 % glycerol), medium purity (80 % to 90 % glycerol), or high purity (above 99 % glycerol). The low- and medium-purity versions are used in animal nutrition and contain 26.8 % and 1.1 % water, 63.3 % and 85.3 % glycerol, and 26.7 % and 0.04 % methanol, respectively (Südekum, 2008). In addition, the source of fiber, source and amount of starch, and NDF and ADF concentrations in experimental diets can affect nutrient intake and digestibility. For example, Lage et al. (2010) and Ribeiro et al. (2018), like this study, used crude glycerin to replace corn in the lambs' diet, which is the main source of starch. For this reason, there was a reduction in the amount of NFC in the diets with the inclusion of glycerin, with a consequent reduction in the consumption of NFC and/or TC. The amounts of NDF in the animals' diet in our trial were similar, and it is probable that this is the reason that we did not observe differences in the intake of this nutrient. The opposite was reported by the aforementioned authors, who, with the inclusion of crude glycerin in the diet, observed a decrease in the concentration of NDF, with a consequent reduction in the intake and digestibility of this nutrient.

Apparent nitrogen balance constitutes an important tool for determining nitrogen use efficiency by ruminants (Thirumalesh and Krishnamoorthy, 2013). The animals fed crude glycerin maintained a positive nitrogen balance, although with no significant differences when compared with those in the control group. Oliveira Filho et al. (2016) also did not report an influence of the inclusion of crude glycerin (0, 28, 55, 82, and 108 $g\;{\mathrm{kg}}^{-1}\;\mathrm{DM}$ sugarcane silage) on the nitrogen balance of the lambs. This probably occurred because there was equilibrium and synchrony of protein and energy sources in the diets, which meant that the protein : energy ratio did not affect nitrogen retention. Although there was an increase in the glycerol concentration with the inclusion of crude glycerin in the lambs' diet, this increase does not seem to have negatively affected the growth of bacteria and, consequently, nitrogen retention, as when the glycerin quantity was elevated, no differences in the retained nitrogen quantity were observed (Oliveira Filho et al., 2016).

Although there was no statistical difference in animal performance results, we observed worsening performance with the increased inclusion of glycerin in the diet, even with increasing days in the feedlot. The difference in feedlot days between lambs that were not fed glycerin and those that were fed with 200 $g\;{\mathrm{kg}}^{-1}\;\mathrm{DM}$ was 10 d. This increase in feedlot days was probably due to a decrease in the DM intake and weight gain. With the inclusion of glycerol in the diet of ruminants, increased propionate in the rumen is observed (Ribeiro et al., 2018); this increases adenosine triphosphate (ATP) production because of its use for glucose production and satiety signaling (Reynolds, 1995) and can contribute to a consequent decrease in the DM intake. Ribeiro et al. (2018) found a decrease in the DM intake, final BW, and ADG of lambs fed diets with glycerin at 70, 140, and 210 $g\;{\mathrm{kg}}^{-1}\;\mathrm{DM}$, indicating the inclusion of up to 4.7 % crude glycerin in the diets. According to Schröder and Südekum (2007), glycerin with different degrees of purity can be included up to 10 % DM in ruminant diets without compromising feed intake or the digestibility of feed components. The results observed in our study suggest the possible inclusion of up to 200 $g\;{\mathrm{kg}}^{-1}\;\mathrm{DM}$ of crude glycerin in lambs' diets, as there were no statistical differences between the treatments in the performance, nitrogen balance, intake, and digestibility of most nutrients evaluated. However, the 10 extra days in the feedlot for lambs fed 200 $g\;{\mathrm{kg}}^{-1}\;\mathrm{DM}$ crude glycerin need to be considered.

## 5 Conclusions

Crude glycerin is an efficient alternative to corn in the diet of feedlot lambs fed with sugarcane as roughage. Although it does not affect the intake and digestibility of most nutrients, the inclusion of 100 and 200 $g\;{\mathrm{kg}}^{-1}\;\mathrm{DM}$ of crude glycerin in the diet tends to worsen lamb performance, indicating that the ideal level of inclusion should be below 100 $g\;{\mathrm{kg}}^{-1}\;\mathrm{DM}$ of crude glycerin.

## Data Availability

The original data used in this study are available from the corresponding author upon request.

## References

[bib1.bib1] AOAC (1998). Official methods of analysis.

[bib1.bib2] Bligh EG, Dyer WJ (1959). A rapid method of total lipid extraction and purification. Can J Biochem Phys.

[bib1.bib3] Borghi TH, Silva Sobrinho AG, Zeola NMBL, Almeida FA, Cirne LGA, Lima ARC (2016). Dietary glycerin does not affect meat quality of Ile de France lambs. Rev Bras Zootecn.

[bib1.bib4] Endo V, Silva Sobrinho AG, Almeida FA, Lima NLL, Zeola NMBL (2015). Digestibility and nitrogen balance of lambs fed sugarcane hydrolyzed under different conditions as roughage in the diet. Cienc Rural.

[bib1.bib5] Gunn PJ, Schultz AF, Van Emon ML, Neary MK, Lemenager RP, Rusk CP, Lake SL (2010). Effects of elevated crude glycerin concentrations on feedlot performance, carcass characteristics and serum metabolite and hormone concentrations in finishing ewe and wether lambs. The Professional Animal Scientist.

[bib1.bib6] Hartman L, Lago RCA (1973). Rapid preparation of fatty acid methyl esters from lipids. Laboratory Practice.

[bib1.bib7] Krehbiel CR (2008). Ruminal and physiological metabolism of glycerin. J Anim Sci.

[bib1.bib8] Lage JF, Paulino PVR, Pereira LGR, Valadares Filho SC, Oliveira AS, Detmann E, Souza NKP, Lima JCM (2010). Crude glycerin on finishing lamb diets. Pesq Agropec Bras.

[bib1.bib9] NRC (National Research Council) (2007). Nutrient Requirements of Small Ruminants: Sheep, Goats, Cervids and New World Camelids.

[bib1.bib10] Oliveira Filho CAA, Azevêdo JAG, Carvalho GGP, Silva CFPG, Cabral ÍS, Pereira LGR, Reis LG, Almeida FM, Souza LL (2016). Crude glycerin combined with sugar cane silage in lamb diets. Trop Anim Health Pro.

[bib1.bib11] Reynolds CK, Englehardt WV, Leonhard-Marek S, Breves G (1995). Ruminant physiology: digestion, metabolism, growth, and reproduction.

[bib1.bib12] Ribeiro RDX, Carvalho GGP, Silva TM, Costa JB, Bezerra LR, Cambuí GB, Barbosa AM, Oliveira RL (2018). Effects of crude glycerin from biodiesel on the diets of lambs: intake, digestibility, performance, feeding behavior, and serum metabolites. J Anim Sci.

[bib1.bib13] Saleem AM, Singer AM (2017). Growth performance and digestion of growing lambs fed diets supplemented with glycerol. Animal.

[bib1.bib14] SAS (Statistical Analysis System) (2009). Version 9.1.

[bib1.bib15] Schröder A, Südekum KH (2007). Glycerol as a byproduct of biodiesel production in diets of ruminants.

[bib1.bib16] Sniffen CJ, Connor JD, Van Soest PJ (1992). A net carbohydrate and protein system for evaluation cattle diets. II Carbohydrate and protein availability. J Anim Sci.

[bib1.bib17] Südekum KH, Garnsworthy PC, Wiseman J (2008). Recent advances in animal nutrition.

[bib1.bib18] Thirumalesh T, Krishnamoorthy U (2013). Rumen Microbial Biomass Synthesis and Its Importance in Ruminant Production. International Journal of Livestock Research.

[bib1.bib19] Van Soest PJ (1994). Nutritional ecology of the ruminant.

